# Age-Related Variations of Genetic and Environmental Contributions to the Covariation of Fear, Distress and Externalizing Symptoms: A Twin Study in Childhood and Adolescence

**DOI:** 10.1007/s10578-023-01498-w

**Published:** 2023-01-25

**Authors:** Stefano De Francesco, Simona Scaini, Guido Alessandri, Emanuela Medda, Laura Camoni, Maria Antonietta Stazi, Corrado Fagnani

**Affiliations:** 1 Child and Youth Lab, Sigmund Freud University, Milan, Italy; 2 Department of Psychology, University of Rome “Sapienza”, Via Dei Marsi 78, 00185 Rome, Italy; 3 Centre for Behavioural Sciences and Mental Health, Istituto Superiore Di Sanità, Rome, Italy

**Keywords:** Twin study, Internalization externalization, Latent susceptibility factor, Children and adolescents

## Abstract

The frequency with which Internalizing and Externalizing symptoms co-occur suggests that, behind both domains, there may be a common susceptibility represented by a general psychopathology factor. However, it’s still unclear whether this common susceptibility is affected by age-related variations. Internalizing (i.e., Fear and Distress) and Externalizing symptoms were evaluated in 803 twin pairs from the population-based Italian Twin Registry. Model-fitting analysis was performed separately in the 6–14 and 15–18 age groups to estimate genetic and environmental contributions to the covariance among symptoms. For the 6–14 group, a multivariate Cholesky model best fitted the data, while, for the 15–18 group, the best fit was provided by a Common Pathway model in which nearly 50% of total variance of each trait was mediated by common genetic factors. Our findings support a common susceptibility behind Internalizing and Externalizing symptoms, mainly genetic in origin, that becomes more evident at the beginning of puberty.

## Introduction

The study of mental disorders in childhood and adolescence is gaining more and more importance in mental health research, given their high prevalence rates in young ages. It has been estimated, in fact, that about 10–20% of young people worldwide are affected by this kind of pathologies, with symptoms that usually persist throughout adulthood [1].

Since the birth of modern psychiatry, mental illnesses have always been categorized as single units, identified on the basis of precise diagnostic criteria (i.e., characteristics and severity of symptoms and their trajectories over time), in order to facilitate the identification of the physical etiological factors specifically linked to each disorder [2].

However, although it has always played a primary role in mental health clinical research and practice over the years, this categorial approach went through numerous criticisms, which started to question its diagnostic reliability. One of the main challenges that this diagnostic system had to face, starting from the DSM-III, was the comorbidity (i.e., co-occurrence) among disorders, a phenomenon that started to be increasingly reported in epidemiological psychiatric studies [3].

The significant frequency of comorbid cases soon led clinicians to reconsider the structure itself of mental disorders, opening to the possibility that these disorders might be read in the light of a more parsimonious model in which they are grouped into macro-categories, rather than considered as independent entities [3]. In one of the first studies that tried to introduce a new interpretation of the pre-existing taxonomic system [4], a confirmatory factor analysis (CFA) was conducted to analyze the correlations among ten of the most common mental disorders, assessed in a probabilistic sample through a structured interview based on DSM-III-R diagnostic criteria. Results showed that the best structural model explaining the comorbidity links among the disorders was indeed a model including three factors, namely Anxious-Misery, Fear and Externalizing, with the first two being highly correlated (*r* = 0.73) and, therefore, possibly reflecting a single higher-order construct, namely the Internalizing domain. It should be noted that, over the years, the label Anxious-Misery has been replaced with Distress, mainly to emphasize the pervasive yet heterogeneous sensation of subjective stress of individuals whose symptoms fall within this category [5]. More specifically, Anxious-Misery and Fear usually refer to symptoms that lead to an impairment that is less evident from the outside, more strictly intrapsychic, while the label Externalizing is commonly used to cluster those behaviors that negatively affect individuals’ interaction patterns with the environment [6].

Further support of this model came from the results of a meta-analytic work [7]. Analyzing the existing literature and comparing the various structural models through model-fitting procedures, the authors showed that a three-factor model provided the best fit to the data. Moreover, they further stressed the division of Internalizing symptoms into the lower-order categories of Distress, in case they were linked to major depression, dysthymia and generalized anxiety disorder, and of Fear, when they were associated to more specific anxiety conditions.

The co-occurrence of disorders within each of the categories composing the three-factor model is a widely known phenomenon [8, 9]. Nevertheless, recent findings in the epidemiological field also showed consistent comorbidity rates between symptoms belonging to different higher-order variables of the structural model, in children-adolescents and in adults, in both clinical and general population samples [8–11].

The observed correlational bond between symptoms belonging to Fear, Distress and Externalizing domains led some researchers to hypothesize that, behind these constructs, there might be a single common susceptibility [8, 11]. In particular, Caspi et al. [8] theorized a structural model of psychopathology consisting of a pyramidal structure. At the basis of the pyramid there would be the disorders conceived as independent entities, while the second step of the structure would be characterized by the Internalizing (hence its lower-order domains) and Externalizing categories that eventually, on the top, would merge into a single continuous dimension of psychopathology, that is the so called “*p* factor”. From a clinical point of view, a higher *p*-score will correspond to a worse assessment of the individual with respect to the severity of the disorder, the sequential comorbidity, the developmental history, and the impairment in adult life [8]. Although this hypothesis has been supported by several subsequent studies, it still remains to be established whether the common liability is a stable phenomenon or it is affected by variations due to age. There is no doubt that the aging process can cause modifications in developmental trajectories of mental disorders [12], with a general trend characterized by a decreasing of Externalizing problems and an increasing of generalized anxiety and depressive symptoms with age [13]. Developmental trajectories of this type of symptoms have also been targeted by twin studies, which allowed to investigate the nature of the factors responsible for their stability and their changes over time. In particular, some of these studies showed that genetic factors underlying the symptoms can not only influence their stability but also contribute to their age-related modifications [14, 15]. The notion that genes exert a continuous influence on certain phenotypes led several authors to ask whether the genetic and environmental structure of Fear, Distress and Externalizing symptoms could justify their inclusion in the three-factor model independently from age. In this respect, the results of a work by Waszczuk et al. showed substantial age-related changes in the genetic influence on Internalizing symptoms. These authors found that, during childhood, there was only one single common genetic factor behind all the anxiety disorders, which gave an irrelevant contribution to the variance of depressive symptoms; instead, common genetic influences underlying both anxiety (especially generalized anxiety disorder) and depression were found starting from adolescence and became more and more evident in adulthood, suggesting the idea that the effect of common biological influences can be observed concurrently with the onset of puberty [16].

Thus, in the light of previous literature mainly regarding the reformulation of structural models of psychopathology, in our study we set two goals: (1) to test the hypothesis of the existence of a common latent susceptibility behind the co-occurrence between Fear, Distress and Externalizing symptoms; (2) to test the stability of the common susceptibility in relation to individuals’ age.

To these aims, a twin sample of children and adolescents of Caucasian origin, living in different Italian regions, was stratified into two age groups, namely 6–14 and 15–18 years, in order to mirror, as best as possible, the division made by Waszczuk et al. [16] in their work.

## Method

### Participants

This study is part of a project involving the population-based Italian Twin Registry (ITR). The procedures that led to the establishment of the ITR are described in detail elsewhere [17]. The Registry currently contains information on more than 29,000 twins from all over Italy, and is extensively exploited for national and international research projects, particularly in the area of behavioral and psychiatric genetics [18]. The sample involved in the present study consisted of 803 twin pairs aged 6–18 years (mean 14.8 ± 2.53 years), with a perfectly balanced gender distribution (~ 50% males). Moreover, at the time of recruitment, none of the participating children carried certified mental/physical handicaps that would require special attention, such as a remedial teacher or differential academic programmes. Twins’ zygosity was determined through the parent-rated Goldsmith questionnaire [19]. According to its algorithm, which generally shows an accuracy of more than 94%, there were 156 monozygotic (MZ) male, 138 MZ female, 271 same-sex dizygotic (DZS, 126 male, 145 female) and 238 unlike-sex dizygotic (DZU) twin pairs in the sample. Prior to model-fitting analyses, the sample was stratified into two age groups: 6–14 years (mean 12.5, 36% MZ, 365 twin pairs), and 15–18 years (mean 16.7, 37% MZ, 438 pairs). In each group the gender distribution mirrored the one of the whole sample (~ 50% males). Table 1 shows demographic characteristics and the mean values of Fear, Distress and Externalizing symptoms in twins considered as individuals and divided by gender, zygosity and age group. All study procedures were accepted by the ethics committee of the Italian National Institute of Health (Istituto Superiore di Sanità). For all participants, parents signed an informed consent.

**Table 1:** Demographic characteristics, and CBCL/6–18 Fear, Distress and Externalizing values, by gender, zygosity and age

	Fear	Distress	Externalizing
	Mean (SD)	Mean (SD)	Mean (SD)
Entire sample (*N* = 1606)	3.1 (6.6)	8.7 (22.9)	11.6 (30)
Males (*N* = 802)	2.9 (6.5)	8 (22.9)	12.4 (30)
Females (*N* = 804)	3.2 (6.7)	9.4 (23.1)	10.8 (30)
	^a^*p* = 0.032	^a^*p* = 0.001	^a^*p* = 0.000
	^b^*p* = 0.773	^b^*p* = 0.046	^b^*p* = 0.394
MZ^c^(*N* = 588)	2.74 (4.8)	6.8 (17.4)	9.4 (22.1)
DZ^d^ (*N* = 1018)	3.3 (7.5)	9.8 (25.6)	12.9 (33.7)
	^a^*p* = 0.969	^a^*p* = 0.017	^a^*p* = 0.084
	^b^*p* = 0.088	^b^*p* = 0.010	^b^*p* = 0.274
Pre-puberty (*N* = 730)	2.9 (6.1)	7.8 (21.4)	11 (27.8)
Post-puberty (*N* = 876)	3.2 (7)	9.4 (24.2)	12.1 (31.7)
	^a^*p* = 0.435	^a^*p* = 0.013	^a^*p* = 0.388
	^b^*p* = 0.557	^b^*p* = 0.171	^b^*p* = 0.952

^a^t-test of means

^b^Levene’s test of variances

^c^Monozygotic

^d^Dizygotic

### Behavioral Measures

The CBCL/6–18 [20] is a standardized questionnaire made up of 118 items through which parents can rate the behavioral and emotional problems of their children on a three-point Likert scale, based on their conduct during the last 6 months. The CBCL/6–18 includes eight Syndromic and six DSM-oriented scales. The first ones were empirically built through exploratory and confirmatory factor analyses; the second ones were obtained by grouping the items in a way that the resulting symptoms in each subscale were consistent with those reported in the DSM-IV. Only the DSM-oriented scales were used in the present study to cluster the symptoms. In particular, items were arranged into the Fear, Distress and Externalizing subscales, mirroring the structure used in a work by Kushner et al. [21]. The Fear variable was composed by the items in the Anxiety Problems scale, the Distress variable was composed by the items in the Somatic Problems and Affective Problems scales, while the Externalizing variable was composed by the items in the Attention Deficit/Hyperactivity Problems, Oppositional Defiant Problems and Conduct Problems scales.

These three broad-band dimensions showed high reliability indexes (Cronbach’s alpha = 0.90 for Fear, 0.95 for Distress and 0.98 for Externalizing) in our sample.

### Preliminary Psychometric Analyses

The hypothesized three-factor model was tested using Exploratory Structural Equation Models (henceforth, ESEM; [22, 23]). ESEM integrates features of confirmatory factor analysis (CFA) and exploratory factor analysis (EFA) allowing researchers to evaluate the fit of alternative theoretical “a priori” models, but relaxing the restrictive “independent clusters assumption” (i.e., all the items have just one loading on the respective factor, and no secondary loadings on different factors; see [24]. In performing the analysis, the dependence of twin data within pairs was taken into account, employing an estimation procedure that “includes a Taylor series-like function to provide a normal theory covariance matrix for analysis” [25] and produces correct parameter estimates, standard errors, and test statistics.

In estimating all the parameters, we used the categorical variable estimator weighted least squares with mean- and variance-adjusted standard errors (WLSMV) over polychoric correlations [26], as implemented in Mplus 8.3 [27]. This estimator is more suited to the ordered-categorical nature of the three-point Likert scale than traditional maximum likelihood estimation [28–30].

Goodness of fit of the model was evaluated by inspecting the WLSMV-based chi-square-statistic, the Comparative Fit Index (CFI), the Tucker Lewis Index (TLI), the Root Mean Square Error of Approximation (RMSEA). Values of RMSEA < 0.06 and CFI-TLI > 0.90 [31] were considered acceptable.

### Exploratory Structural Equation Modeling Analysis

The fit of the hypothesized three-factor ESEM model was acceptable, with χ^2^(1323, *N* = 1590) = 2677.36, *p* < 0.001; CFI = 0.918; TLI = 0.908; RMSEA = 0.025, 90% CI [0.024, 0.027], *p* = 1.00. All primary factor loadings were significant and, on average > 0.30, with a mean of 0.40 (SD = 0.18) for Fear, 0.43 (SD = 0.21) for Distress, and 0.65 (SD = 0.15) for Externalizing. Secondary loadings had a mean of 0.07 (SD = 0.25) for Fear, 0.35 (SD = 0.24) for Distress, and 0.12 (SD = 0.15) for Externalizing. Among secondary loadings, 13 for Fear, 10 for Distress, and 8 for Externalizing out of 50 resulted not statistically significant. Correlations among latent factors ranged from low (0.05) but statistically significant (*p* = 0.015) between Fear and Distress, to moderate and significant between Fear and Externalizing (0.32, *p* < 0.001), and between Distress and Externalizing (0.28, *p* < 0.001).

### Data analyses

All the twin analyses were performed using the Mx package [32].

First, the multivariate correlation pattern was estimated by means of phenotypic correlations (i.e., between different subscales within a twin individual), cross-twin/within-trait correlations (i.e., between twin and cotwin for the same subscale, separately for MZ and DZ pairs) and cross-twin/cross-trait correlations (i.e., between a given subscale in a twin and a different subscale in the cotwin, separately for MZ and DZ pairs). These correlations are informative about the genetic and environmental effects on variance and covariance of the analysed phenotypes, and were estimated using a saturated model with the following constraints: (i) same means and variances of Fear, Distress and Externalizing for twin1 and twin2, MZ and DZ, based on the assumption that twins, as individuals, are representative of the general population; (ii) same cross-twin/cross-trait covariances regardless of twin order (i.e., covariance between trait-x in twin1 and trait-y in twin2 equal to covariance of trait-y in twin1 and trait-x in twin2) within each zygosity group, assuming symmetry between twins of the same pair. Second, multivariate structural equation twin models were applied to estimate genetic and environmental contributions to variance and covariance of the traits. More precisely, multivariate twin designs allow to decompose variance and covariance of the traits into contributions due to additive genetic factors (A) (i.e., additive effects of all gene variants influencing the traits, without interactive effects), common environmental factors (C) [i.e., effects of environmental factors that are shared by the twins within the family, particularly during childhood and adolescence (e.g., rearing environment, family socio-economic status, parental behaviours, etc.), or that are shared in the womb (e.g., hormonal exposures)], and unique (individual-specific) environmental factors (E) [i.e., effects of environmental factors that specifically act on an individual (e.g., lifestyles, relations with peers, infections, etc.), including measurement error]. Different multivariate models, namely the Cholesky model, the Independent Pathway model, and the Common Pathway model, were applied to detect the best representation of data in each age group separately. The Cholesky model represents the association among phenotypes through common latent factors. For *n* variables, a Cholesky decomposition includes *n* independent genetic and environmental factors: the first factor loads on all traits, the second one loads on all traits but the first, the third factor loads on all traits but the first two, and so on [10, 33].

The Independent Pathway model assumes that common genetic and environmental latent variables exert a direct influence on all the phenotypes included in the design. These common factors would account for the covariance among the traits, while a set of specific latent variables would be responsible for the unshared variance of each trait [34].

In the Common Pathway model, the covariance among phenotypes depends on a single common latent factor that exerts a direct influence on each observed variable, and its variance is determined by genetic and environmental factors. Moreover, the model includes trait-specific latent factors that represent the unshared portions of variance [35].

All models were compared to each other, both in their full version and in their reduced form. Model comparison was performed via chi-square (χ^2^) tests, and the selection of the best-fitting model was guided by the principle of parsimony. According to this principle, models with fewer latent variables were preferred over the more complete ones if they didn’t cause a significant worsening of fit to the data. This was accomplished by selecting those models showing the lowest Akaike Information Criterion (AIC) and, at the same time, a non-significant χ^2^ test.

Prior to data analyses, all the scales were log-transformed to approximate normal distributions. Age and gender were included as covariates in all models.

## Results

Table 1 reports mean values of Fear, Distress and Externalizing scales in twins considered as individuals and divided by gender, zygosity and age group. Comparisons by t-tests showed higher scores in Fear and Distress symptoms for girls compared to boys, with an opposite pattern for Externalizing symptoms. Age-related differences in mean scores were found only for Distress symptoms, higher in the 15–18 group, while no zygosity differences were detected.

### Age group 6–14

**Fig. 1:**
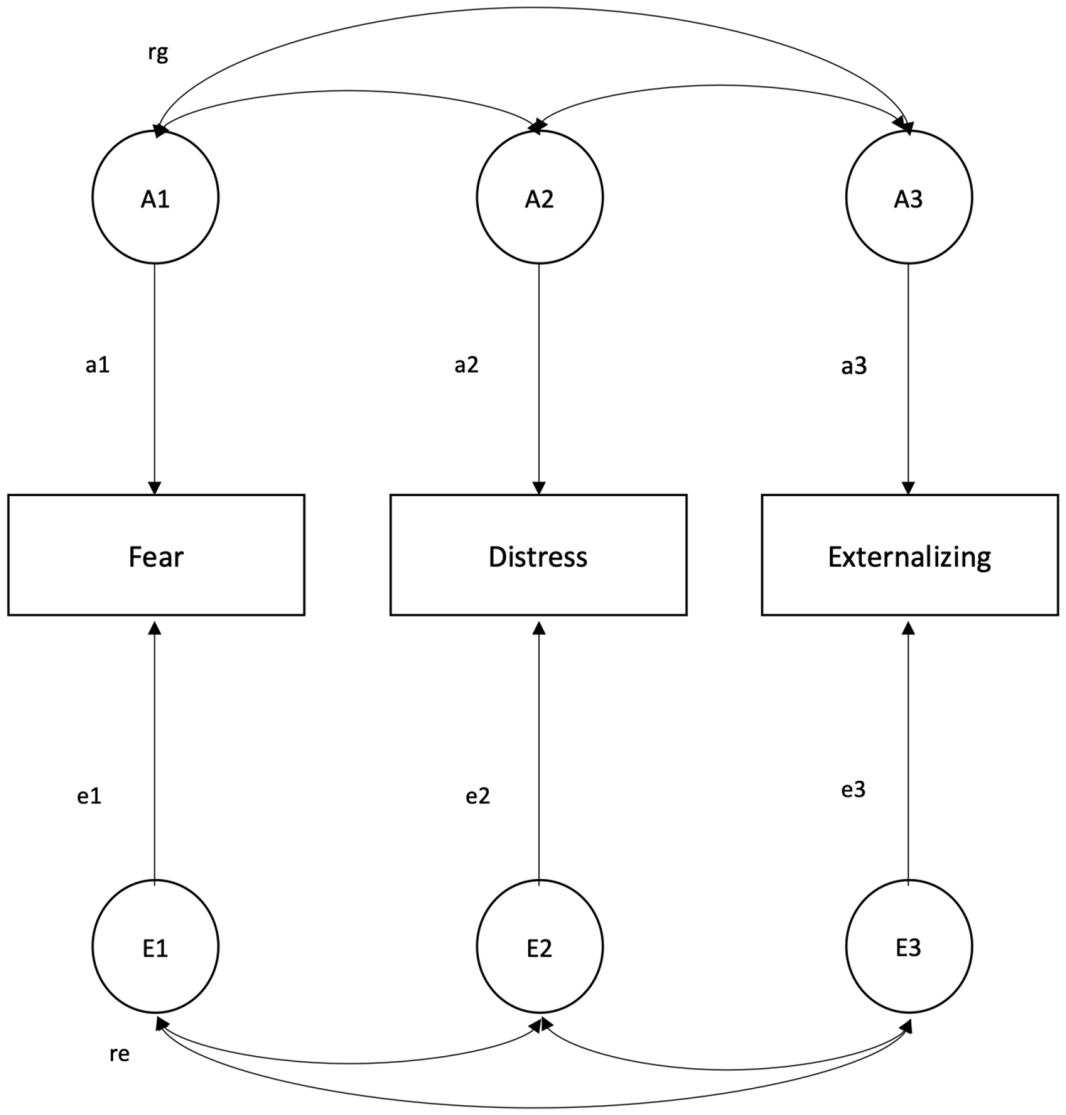
Cholesky AE model

Table 2 shows the correlation pattern for the 6–14 age group. Substantial phenotypic correlations among the scales were found, and cross-twin/within-trait correlations were higher in MZ than in DZ pairs, consistent with genetic influences on the phenotypes. Cross-twin/cross-trait correlations were also higher in MZ pairs, suggesting genetic effects shared by the traits. Table 3 shows the results of model-fitting analyses and model comparisons. The best-fitting model (i.e., the one with the lowest AIC that didn’t produce a significant worsening of fit) was the Cholesky AE (model 2) encompassing only additive genetic and unique environmental latent sources (Fig. 1). Genetic and unique environmental contributions to the traits, obtained from the best-fitting model, are reported in Table 4. The genetic proportions of variance were similar across the phenotypes, with all heritability estimates close to 0.50 and a slightly higher value for Externalizing symptoms. Regarding the covariance among the scales, it was mainly explained by additive genetic factors, with proportions ranging from 62 to 83%. Genetic correlations suggested a considerable overlap of additive genetic factors influencing the different traits, and a much weaker unique environmental overlap.

**Table 2:** Correlations in the 6–14 age group

Phenotypic correlations			
	Fear (2.9 ± 6.1)	Distress (7.8 ± 21.4)	Externalizing (11 ± 27.8)
Fear	1	–	–
Distress	.56	1	–
Externalizing	.59	.56	1

Cross-twin/within-trait correlations			
	Fear (2.9 ± 6.1)	Distress (7.8 ± 21.4)	Externalizing (11 ± 27.8)
MZ^a^	.50	.51	.57
DZ^b^	.33	.34	.28

Cross-twin/cross-trait correlations			
MZ^a^	Fear (2.9 ± 6.1)	Distress (7.8 ± 21.4)	Externalizing (11 ± 27.8)
Fear	1	–	–
Distress	.32	1	–
Externalizing	.47	.39	1

DZ^b^	Fear (2.9 ± 6.1)	Distress (7.8 ± 21.4)	Externalizing (11 ± 27.8)
Fear	1	–	–
Distress	.24	1	–
Externalizing	.29	.27	1

Mean and standard deviation of each scale are presented in parentheses

^a^Monozygotic

^b^Dizygotic

**Table 3:** Model-fitting analyses and model comparisons in the 6–14 age group

Model	c.t.m.^c^	− 2LL^a^	DF^b^	AIC^g^	χ^2 d^	ΔDF^e^	*p*^f^
1. Cholesky A^h^ C ^i^ E^j^	–	− 549.73	2158	− 3766.27	–	–	–
2. Cholesky A^h^ E^j^	**1**	− **552.91**	**2164**	− **3775.09**	**3.180**	**6**	**0.786**
3. Common Pathway A^h^ C ^i^ E^j^	–	− 559.56	2162	− 3764.43	–	–	–
4. Common Pathway A^h^ E^j^	3	− 562.34	2166	− 3769.65	2.783	4	0.595
5. Independent Pathway A^h^ C ^i^ E^j^	–	− 550.31	2158	− 3765.68	–	–	–
6. Independent Pathway A^h^ E^j^	5	− 553.02	2164	− 3774.97	2.710	6	0.844

Best-fitting model is printed in boldface type

^a^minus twice the log-likelihood

^b^degrees of freedom

^c^compared to model

^d^(− 2LL submodel)–(− 2LL full model)

^e^(DF submodel)–(DF full model)

^f^probability

^g^ − 2LL − 2**DF**

^h^additive genetic influence

^i^shared environmental influence

^j^unshared environmental influence

**Table 4:** Genetic and environmental variance–covariance components and correlations of CBCL/6–18 Fear, Distress and Externalizing behaviors, as estimated from the best-fitting Cholesky AE model for the 6–14 age group

Standardized components			
	A^g^	C^h^	E^i^
Vp (Fear)^a^	.54 (.42–.63)	–	.46 (.37–.57)
Vp (Distress)^b^	.54 (.43–.63)	–	.46 (.37–.57)
Vp (Est-Dos)^c^	.56 (.45–.65)	–	.44 (.35–.55)
Cov (Fear-Distress)^d^	.62 (.47–.75)	–	.38 (.25–.53)
Cov (Fear-Est-Dos)^e^	.83 (.69–.94)	–	.17 (.06–.31)
Cov (Distress-Est-Dos)^f^	.74 (.60–.86)	–	.26 (.14–.40)

Genetic and environmental correlations			
	rg^j^	rc^k^	re^l^
Fear-Distress	.65 (.53–.76)	–	.46 (.33–.56)
Fear-Est-Dos	.89 (.78–1)	–	.23 (.09–.36)
Distress-Est-Dos	.76 (.64–.86)	–	.32 (.19–.44)

Number in parentheses are 95% confidence intervals

^a^phenotypic variance of Fear behaviors

^b^phenotypic variance of Distress behaviors

^c^phenotypic variance of Externalizing behaviors

^d^covariance between Fear and Distress behaviors

^e^covariance between Fear and Externalizing behaviors

^f^covariance between Distress and Externalizing behaviors

^g^additive genetic influence

^h^shared environmental influence

^i^unique environmental influence

^j^additive genetic correlation

^k^shared environmental correlation

^l^unique environmental correlation

### Age group 15–18

**Fig. 2:**
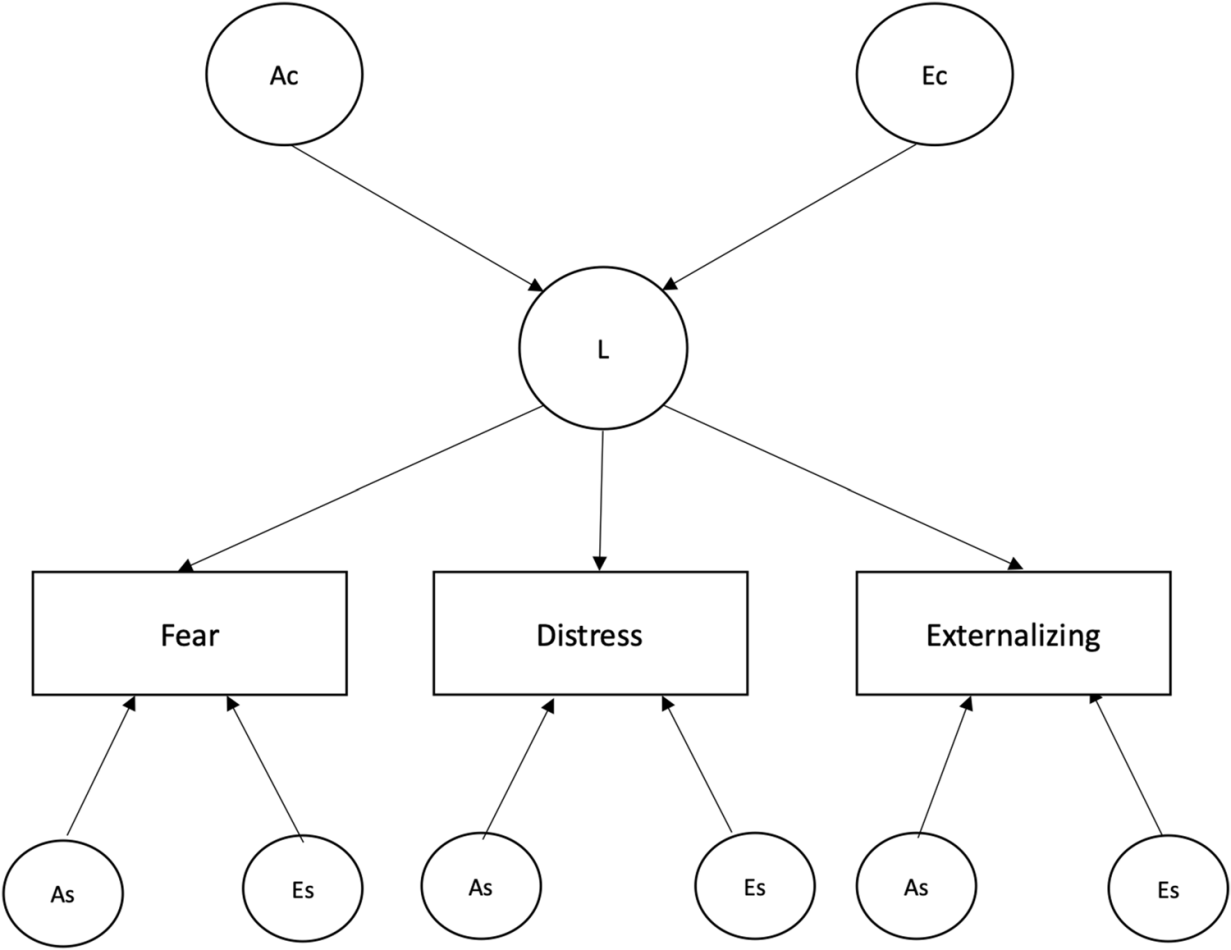
Common pathway model AE

Phenotypic correlations in the subgroup aged 15–18 were slightly higher than those found in the younger age group, with estimates above or equal to 0.60 (Table 5). Moreover, the twin correlation pattern suggested, also in this case, genetic effects on variance and covariance of all traits. Results of model-fitting analyses are shown in Table 6. The best-fitting model was the Common Pathway AE (model 4), depicted in Fig. 2. Under this model, the common latent susceptibility factor was largely genetically influenced, with a heritability estimate of 0.77. Table 7 reports additive genetic and unique environmental contributions to the variance of the traits, as derived from the best-fitting model. For all the phenotypes, heritability was 0.60, and a large portion of it (estimated proportions of variance from 0.44 to 0.49; hence, estimated proportions of heritability from 73 to 82%) was explained by genetic factors shared by the phenotypes.

**Table 5:** Correlations in the 15–18 age group

Phenotypic correlations			
	Fear (3.2 ± 7)	Distress (9.4 ± 24.2)	Externalizing (12.1 ± 31.7)
Fear	1	–	–
Distress	.61	1	–
Externalizing	.63	.60	1

Within-twin/cross-trait correlations			
	Fear (3.2 ± 7)	Distress (9.4 ± 24.2)	Externalizing (12.1 ± 31.7)
MZ	.62	.53	.60
DZ	.34	.37	.36

Cross-twin/cross-trait correlations			
MZ^a^	Fear (3.2 ± 7)	Distress (9.4 ± 24.2)	Externalizing (12.1 ± 31.7)
Fear	1	–	–
Distress	.46	1	–
Externalizing	.49	.43	1

DZ^b^	Fear (3.2 ± 7)	Distress (9.4 ± 24.2)	Externalizing (12.1 ± 31.7)
Fear	1	–	–
Distress	.25	1	–
Externalizing	.32	.26	1

Mean and standard deviation of each scale are presented in parentheses

^a^Monozygotic

^b^Dizygotic

**Table 6:** Model-fitting analyses and model comparisons in the 15–18 age group

Model	c.t.m.^c^	− 2LL^a^	DF^b^	AIC^g^	χ^2 d^	ΔDF^e^	*p*^f^
1. Cholesky A^h^ C^i^ E^j^	–	− 699.49	2597	− 4494.50	–	–	–
2. Cholesky A^h^ E^j^	1	− 707.56	2603	− 4498.43	8.070	6	0.233
3. Common pathway A^h^ C^i^ E^j^	–	− 703.54	2601	− 4498.45	–	–	–
**4. Common pathway** A^h^ E^j^	**3**	− **710.34**	**2605**	− **4499.65**	**6.798**	**4**	**0.147**
5. Independent pathway A^h^ C^i^ E^j^	–	− 699.94	2597	− 4494.05	–	–	–
6. Independent pathway A^h^ E^j^	5	− 707.56	2603	− 4498.43	7.623	6	0.267

Best-fitting model is printed in boldface type

^a^minus twice the log-likelihood

^b^degrees of freedom

^c^compared to model

^d^(−2LL submodel)—(−2LL full model)

^e^(DF submodel)—(DF full model)

^f^probability

^g^ − 2LL − 2**DF**

^h^additive genetic influence

^i^shared environmental influence

^j^unshared environmental influence

**Table 7:** Genetic and environmental proportions of variance of CBCL/6–18 Fear, Distress and Externalizing behaviors, as estimated under the best-fitting Common Pathway AE model for the 15–18 age group

	A_C_^a^	A_S_^b^	E_C_^c^	E_S_^d^	a2^e^	e2^f^
Fear	.49 (.41–.57)	.11 (.04–.18)	.15 (.10–.21)	.25 (.19–.32)	.60 (.52–.68)	.40 (.32–.48)
Distress	.44 (.38–.52)	.16 (.09–.22)	.13 (.09–.19)	.27 (.20–.33)	.60 (.52–.67)	.40 (.33–.48)
Externalizing	.47 (.39–.54)	.13 (.06–.20)	.14 (.1–.20)	.26 (.20–.32)	.60 (.52–.68)	.40 (.32–.48)

Number in parentheses are 95% confidence intervals

^a^Additive genetic effects common to the traits (i.e., mediated by the common susceptibility factor)

^b^phenotype-specific additive genetic effects

^c^unique environmental effects common to the traits

^d^phenotype-specific unique environmental effects

^e^total additive genetic effects (i.e., total heritability)

^f^total unique environmental effects

## Discussion

Our results supported the co-occurrence between Internalizing (i.e., Fear-Distress) and Externalizing symptoms, with substantial correlation rates among the three analyzed phenotypes in the 6–14 and 15–18 age groups. This was consistent with previous studies of psychopathology in childhood and adolescence [8–11], and was in line with available theories on the comorbidity bond existing between these diagnostic domains [3]. Multivariate model-fitting analyses showed a null contribution of shared environment on all the traits in both age groups. Although this finding in the 15–18 age group might not seem surprising, the lack of evidence of common environmental effects in the younger group should be discussed, as the influence of shared environment tends to play a predominant role during childhood [36]. One possible explanation could be that the 6–14 age group may not be strictly representative of childhood age, as it includes adolescents of 11–14 years who may have caused a considerable reduction in the shared (familial) influence on the traits. To test this hypothesis, we further stratified our sample by age, identifying three subsamples, namely 6–10, 11–14 and 15–18 years old. As expected, cross-twin/within-trait correlational trends obtained in the three subsamples separately did unravel a possible role of common environment on the traits during infancy, with lower values of correlations in the 6–10 MZ group for all the three phenotypes (data not shown).

With respect to the main aim of our study, results from multivariate modelling suggested the existence of a common susceptibility factor behind the co-occurrence between Fear, Distress and Externalizing symptoms, which, however, seemed to become evident only starting from adolescence. In fact, the best-fitting models found in the two age groups were indicative of an age-related variation of the genetic and environmental influences on the phenotypes. More specifically, the Cholesky AE model that best fitted the data in the 6–14 age group suggested that, although highly correlated among the traits, genetic and environmental influences on Fear, Distress and Externalizing symptoms may still act through distinct patterns at this age. On the other hand, the best-fitting Common Pathway AE model that was found in the 15–18 age group indicated that the etiological influences on all the phenotypes may begin to acquire a common nature over time. These findings are consistent with those of Waszczuk et al. [16], who considered only Internalizing-like symptoms, and suggested a delay in the effect of new genetic influences coming into play with puberty. Thus, our study would show that the same pattern of etiological variation, with a similar interpretation as in the above-mentioned work, might hold when Externalizing symptoms are included in the experimental design, beside the Internalizing dimensions of Fear and Distress. Our finding on common etiological substrates behind the co-occurrence of the three symptoms domains is consistent with the hypothesis on the existence of a unique latent factor predisposing to psychopathology [8]. Similar evidence supporting the “*p* factor” of psychopathology can be found in Allegrini et al. [37]; in this study, authors reported a common latent variable, underlying the targeted symptoms, that was heritable at 50–60% [37]. A highly interesting consideration about the phenotypic nature of the general psychopathology factor relates this factor to personality traits [3, 37, 38]. In fact, it has been reported by several studies that many childhood disorders belonging both to the internalizing and the externalizing domain might be more faithfully described in terms of personality traits rather than categorical dimensions [39]. From a structural point of view, personality is constituted by two major domains: temperament and character [40]. Temperament can be defined as the portion of personality that is highly heritable, while character is mainly influenced by the environment. Personality is the result of the constant interaction of these two domains, which extensively occurs during development. That said, it appears clear how much personality can be regarded as something that is strictly connected to the neurobiological substratum of individuals, hence to their genes [40]. In the light of this, the most recent psychiatric genetics findings on the highly genetic nature of the general factor of psychopathology undoubtably highlight the importance of taking into account personality when theorizing a structural model of mental disorders that aims to be as accurate as possible [41]. From a developmental perspective, personality traits tend to increase in maturity with age. More specifically, previous studies have found that functional traits, such as conscientiousness and agreeableness, normally increase, whereas dysfunctional ones, such as neuroticism, tend to decrease over time [42]. Nevertheless, during early adolescence, this tendency towards improvement can change in the opposite direction, causing a temporary decline in personality maturation [42]. Adolescence is considered one of the most critical periods for personality development [42, 43], as it is also the usual onset age of personality disorders when in conjunction with particular circumstances, such as Internalizing or Externalizing disorders not properly treated [43, 44]. One of the main tasks of adolescence is, in fact, the development of one’s own identity, mainly through the social comparison with peers as a mean of self-evaluation [43]. From a neurobiological standpoint, this process is supported by the dorsal medial prefrontal cortex (MPFC), a brain region that starts developing during adolescence, and that allows the interpretation of social stimuli in a self-reflective key, making the experience of self-conscious emotions and autonomic arousal possible [43]. Our results seem consistent with this mechanism, as they suggest that the (highly genetically determined) influence of the latent susceptibility, common to Fear, Distress and Externalizing symptoms, may come effectively on-line during adolescence. Trying to be more specific about the psychological nature of the latent factor, numerous studies have identified neuroticism as the most likely personality trait related to Internalizing and Externalizing problems [45, 46]. Neuroticism (or negative affect) is defined as the tendency to experience frequent negative emotions, such as anger, sadness, guilt, and nervousness [47], which usually leads to frequent worry, emotional avoidance, and rumination [48]. High levels of neuroticism could lead to biased interpretations of the social stimuli, making people perceive ordinary situations as unreasonably threatening [49]. A very intriguing hypothesis, deriving from several twin studies that found a significant genetic and environmental overlap of neuroticism with Internalizing and Externalizing problems, suggests that the unique latent susceptibility factor laying behind the comorbidity between these clusters of symptoms might be represented by neuroticism itself [50–52]. In the light of the developmental trajectories of this dispositional trait, our results could be consistent with this hypothesis; in fact, like other dysfunctional personality traits, neuroticism seems to increase during adolescence, as it has been recently reported for girls [42], and this could explain why, in our study, the latent factor becomes more evident during this age. Some limitations should be taken into account when interpreting our results. First, we used only CBCL/6–18 to assess symptoms. Even though this instrument has proved to be highly reliable in both clinical and non-clinical populations at a multicultural level [20, 53], it can be affected by some measurement biases due to its being a parent-rated questionnaire. Among these systematic errors, the most common is usually an overestimation of the Externalizing symptoms and an underestimation of the Internalizing ones [6]; furthermore, in the specific context of twin studies, the use of CBCL/6–18 to assess the symptoms often leads to detect higher levels of the shared environment contribution to the total variance [54]. In the light of this, although this last bias does not seem to have affected our results, the use and comparison of multiple tests in the assessment of symptoms would have increased results’ reliability. Second, problem behaviors were assessed at one time point only; this is a potential confounder for the estimation of the effects of unique environmental factors and for the distinction of these effects from measurement error [10]. Third, even though the sex-stratified correlational pattern didn’t seem to suggest any role of sex in the gene-environment structure of the single traits and their mutual relationships (data not shown), the relatively small size of our sample conferred limited power to formally address potential sex differences in the etiology of symptoms. The importance of this last limitation stems from the observation that developmental trajectories of neuroticism appear to be highly influenced by gender [42], and that there are marked differences in the prevalence rates of internalizing and externalizing problems, which have also been explained in terms of gender-linked behavioral preferences that drive the expression of the individual’s susceptibility toward general psychopathology [8, 55].

## Summary

In conclusion, though with some limitations, the present study supports the existence of a common latent susceptibility factor behind Fear, Distress and Externalizing symptoms. This factor is likely to be mainly genetic in origin, and its effects seem to become more evident during puberty. These findings support the idea that diagnostic decisions are to be made considering the complexity that characterizes the psychological profile of the single individual, including possible comorbidity patterns [39].

In this perspective, results of the present work could be of use in the clinical setting, by encouraging clinicians to monitor the symptomatic manifestations of Externalizing problems both at individual and familial level, when in presence of a patient who comes to clinical attention for Internalizing-like problems, and vice versa. Moreover, the idea that the common etiological factor behind these clusters of symptoms might be represented by neuroticism could bring a new insight even regarding the most effective psychological treatment to use. In this regard, transdiagnostic therapies could be an intriguing and promising approach, aiming to target neuroticism itself rather than the single disorders as fragmented categories [49, 56].
